# Tumor-suppressive *microRNA-29a* inhibits cancer cell migration and invasion via targeting *HSP47* in cervical squamous cell carcinoma

**DOI:** 10.3892/ijo.2013.2145

**Published:** 2013-10-18

**Authors:** NORIKO YAMAMOTO, TAKASHI KINOSHITA, NIJIRO NOHATA, HIROFUMI YOSHINO, TOSHIHIKO ITESAKO, LISA FUJIMURA, AKIRA MITSUHASHI, HIROKAZU USUI, HIDEKI ENOKIDA, MASAYUKI NAKAGAWA, MAKIO SHOZU, NAOHIKO SEKI

**Affiliations:** 1 Departments of Functional Genomics, Chiba University Graduate School of Medicine, Chiba;; 2 Reproductive Medicine, Chiba University Graduate School of Medicine, Chiba;; 3 Department of Urology, Graduate School of Medical and Dental Sciences, Kagoshima University, Kagoshima;; 4 Biomedical Research Center, Chiba University, Chiba, Japan

**Keywords:** *miR-29a*, tumor suppressor, cervical cancer, *HSP47*, migration, invasion

## Abstract

Our recent studies of microRNA (miRNA) expression signatures indicated that *microRNA-29a (miR-29a)* was significantly downregulated in several types of human cancers, suggesting that *miR-29a* may be a putative tumor-suppressive miRNA in human cancers. The aim of this study was to investigate the functional significance of *miR-29a* in cervical squamous cell carcinoma (SCC) and to identify novel *miR-29a*-regulated cancer pathways and target genes involved in cervical SCC oncogenesis and metastasis. Restoration of *miR-29a* in cervical cancer cell lines (CaSKi, HeLa, ME180 and Yumoto) revealed that this miRNA significantly inhibited cancer cell migration and invasion. Gene expression data and *in silico* analysis demonstrated that heat-shock protein 47 *(HSP47),* a member of the serpin superfamily of serine proteinase inhibitors and a molecular chaperone involved in the maturation of collagen molecules, was a potential target of *miR-29a* regulation. Luciferase reporter assays showed that *miR-29a* directly regulated *HSP47.* Moreover, silencing of the *HSP47* gene significantly inhibited cell migration and invasion in cancer cells and the expression of HSP47 was upregulated in cancer tissues and cervical intraepithelial neoplasia (CIN), as demonstrated by immunostaining. Downregulation of *miR-29a* was a frequent event in cervical SCC and *miR-29a* acted as a tumor suppressor by directly targeting *HSP47.* Recognition of tumor-suppressive miRNA-regulated molecular targets provides new insights into the potential mechanisms of cervical SCC oncogenesis and metastasis and suggests novel therapeutic strategies for treatment of this disease.

## Introduction

Cervical cancer is the second most common cause of cancer death in women worldwide and ∼500,000 new cases of cervical cancer are diagnosed each year, with 280,000 deaths (1). Cervical squamous cell carcinoma (SCC) is the most frequent type of cervical cancer and the most important risk factor for cervical-SCC is persistent human papilloma virus (HPV) infection (2–4). High-risk HPVs contain oncoproteins, i.e., E6 and E7, which contribute to the oncogenesis of cervical SCC by silencing tumor-suppressive p53 and Rb proteins and several cancer-related genes (5). Therefore, recent research on cervical SCC has focused on E6 and E7 oncoproteins. However, the molecular mechanisms of cervical SCC initiation, development, and metastasis have not yet been fully elucidated.

The discovery of non-coding RNAs in the human genome was an important conceptual breakthrough in the post-genome sequencing era (6). A growing body of evidence indicates that miRNAs are key regulators that contribute to the initiation and development of various types of cancer (7). In cancer pathways, normal regulatory mechanisms are disrupted by altered expression of tumor-suppressive or oncogenic miRNAs. Therefore, identification of differentially expressed miRNAs is an important step to understanding human oncogenesis.

Based on this, our research group has elucidated the miRNA expression signatures of various types of human cancers (8–12). Recent studies of miRNA expression signatures of hypopharyngeal SCC and maxillary SCC have indicated that expression of *miRNA-29* family miRNAs *(miR-29a/b/c)* is significantly reduced in cancer tissues, suggesting that these miRNAs may contribute to the oncogenesis and metastasis of cervical SCC (13,14).

Expression analysis of *miR-29* family miRNAs in cervical SCC clinical specimens showed that *miR-29a* was the most highly downregulated miRNA in the clinical specimens, thus, we focused on *miR-29a* in this study. The aim of the present study was to investigate the functional significance of *miR-29a* and to identify the molecular target genes regulated by *miR-29a* in cervical SCC cells. Genome-wide gene expression data and *in silico* database analysis showed that the heat-shock protein 47 *(HSP47)* gene, also known as serpin peptidase inhibitor clade H, member 1 *(SERPINH1),* was a promising candidate target of *miR-29a.*

## Materials and methods

### Clinical specimens

A total of 18 primary cervical SCC specimens and 11 non-cancer specimens were collected from patients who had undergone surgical treatment at Chiba University Hospital. The samples were processed and stored in liquid nitrogen until RNA extraction. Patient information is summarized in Table I. Our study was approved by the Bioethics Committee of Chiba University; prior written informed consent and approval was given by each patient. HPV status was examined by L1 consensus primers and type-specific real-time PCR primers, as described previously (15).

### RNA isolation

Total RNA was isolated using TRIzol reagent (Invitrogen, Carlsbad, CA, USA) according to the manufacturer’s protocol. RNA concentrations were determined spectrophotometrically. RNA quality was confirmed using a NanoDrop 1000 Spectrophotometer (Thermo Fisher Scientific, USA).

### Quantitative real-time RT-PCR

Stem-loop RT-PCR (TaqMan MicroRNA assays; P/N, 002112 for *miR-29a;* P/N, 000413 for *miR-29b;* P/N, 000587 for *miR-29c;* Applied Biosystems, Foster City, CA, USA) was used to quantify miRNAs according to earlier published conditions (14). To normalize the data for quantification of *miR-29*-family sequences, we used *RNU48* (Assay ID, 001006; Applied Biosystems) as a control. The ΔΔCt method was used to calculate the fold-change.

### Mature miRNA and siRNA transfections

Cervical cancer cell lines were transfected with Lipofectamine RNAiMAX transfection reagent (Invitrogen) and Opti-MEM (Invitrogen) with 10 nM mature miRNA or siRNA molecules. The following RNA species were used in this study: mature miRNA, mirVana miRNA mimic for *hsa-miR-29a-3p* (Product ID, MC12499; Applied Biosystems), negative control miRNA (P/N, AM17111; Applied Biosystems), small-interfering RNA (Stealth siRNAs, si-SERPINHl; P/N, HSS101423 and HSS189522; Invitrogen) and negative control siRNA (Stealth RNAi Negative Control Medium GC, P/N, 12935-300; Invitrogen).

### Cell proliferation, migration and invasion assays

Cell proliferation was determined using XTT assays (Roche Applied Science, Tokyo, Japan) according to the manufacturer’s instructions. Cell migration assays were performed using modified Boyden Chambers (Transwells, Corning/Costar no. 3422, USA). Cells were transfected with 10 nM miRNA by reverse transfection and plated in 10-cm dishes at 8×l0^5^ cells/dish. After 48 h, 1×10^5^ cells were added to the upper chamber of each migration well and were allowed to migrate for 48 h. After gentle removal of the non-migratory cells from the filter surface of the upper chamber, the cells that migrated to the lower side were fixed and stained with Diff-Quick (Sysmex Corp., Japan). The number of cells migrating to the lower surface was determined microscopically by counting four areas of constant size per well. Cell invasion assays were carried out using modified Boyden chambers in 24-well tissue culture plates at 1×10^5^ cells per well (BD Biosciences, USA). All experiments were performed in duplicate.

### Target gene search for miR-29a

A genome-wide screen was performed to identify *miR-29a*-target genes using *miR-29a*-transfected CaSKi cells. A SurePrint G3 Human GE 8×60K Microarray (Agilent Technologies, Santa Clara, CA, USA) was used for expression profiling of *miR-29a* transfectants in comparison with miRNA-negative control transfectants. TargetScan release 6.2 (http://www.targetscan.org/) was used to identify predicted target genes and their miRNA binding site seed regions. Gene expression data for clinical cervical SCC specimens were obtained from the GEO database (accession no. GSE6791).

### Western blot analysis

Cells were harvested and lysed 72 h after transfection. Each cell lysate (50 *μg* of protein) was separated using Mini-Protean TGX gels (Bio-Rad, Hercules, CA, USA), followed by subsequent transfer to PVDF membranes. Immunoblotting was performed with polyclonal anti-HSP47 antibodies (sc-5293; Santa Cruz Biotechnology, Santa Cruz, CA, USA). Anti-GAPDH antibodies (ab8245; Abeam, UK) were used as an internal control.

### Plasmid construction and dual-luciferase reporter assays

Partial sequences (191 bp) of the *HSP47* 3′ untranslated region (3′UTR) that contain the *miR-29a* target site (GGTGCTA) were inserted between the *Xho*I and *Pme*I restriction sites in the 3′UTR of the hRluc gene in the psiCHECK-2 vector (Promega, Madison, WI, USA). The deletion of the *miR-29a* target site was cloned and constructed as deletion-vector in this study. HeLa cells were then transfected with 5 ng vector and 10 nM mature miRNA.

### Immunohistochemistry

We performed immunostaining using a tissue microarray containing 60 specimens: 10 normal cervical tissues, 10 inflammation tissues, 10 cervical intraepithelial neoplasia (CIN) tissues and 30 SCC tissues (CR 602; US Biomax, Rockville, MD, USA). Detailed information on all tumor specimens can be found at http://www.biomax.us/tissue-arrays/Uterus/CR602. The tissue microarray was incubated overnight with primary mouse monoclonal antibodies against HSP47 (1:50, sc-5293, Santa Cruz Biotechnology). Next, the sample was treated with anti-mouse biotin antibodies (1:2,000, 115-065-003, Jackson ImmunoResearch Laboratories, Inc., West Grove, PA, USA) for 1 h and then treated with an ABC kit (K0377, Dako, Carpinteria, CA, USA) for 30 min. Counterstaining was performed using a DAB kit (425011, Nichirei Bioscience Inc., Tokyo, Japan). Immunostaining was evaluated according to previously described scoring methods (12).

### Statistical analysis

The relationships between two variables and numerical values were analyzed using the Mann-Whitney U test and the relationships between three variables and numerical values were analyzed using the Bonferroni-adjusted Mann-Whitney U test. Expert StatView analysis software (ver. 4; SAS Institute Inc., Cary, NC, USA) was used in both analyses. In the comparison of three variables, an unadjusted statistical level of significance of P<0.05 corresponded to the Bonferroni-adjusted level of P<0.0167.

## Results

### Expression of miR-29-family miRNAs in clinical cervical SCC specimens

**Figure 1.**
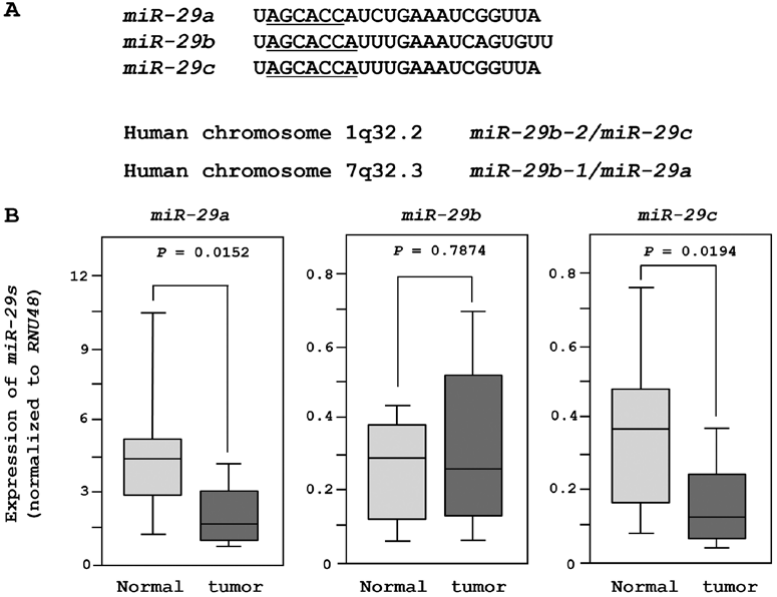
(A) The sequences and chromosomal locations of *miR-29*-family miRNAs. Seed sequences are shown by underlining. (B) Expression levels of *miR-29a/b/c* in cervical SCC tumor tissues and normal tissues, as determined by stem-loop RT-PCR. *RNU48* was used as an internal control.

The sequences and chromosomal locations of *miR-29*-family miRNAs *(miR-29a/b/c)* in the human genome are shown in Fig. 1A. These miRNAs were clustered at two different human genomic loci, *miR-29b-1* and *miR-29a* at 7q32.3 and *miR-29b-2* and *miR-29c* at lq32.2.

We evaluated the expression of *miR-29*-family miRNAs in 18 clinical specimens and 11 non-cancer tissues. The expression levels of *miR-29a* and *miR-29c* were significantly lower in tumor tissues than in non-cancer tissues. However, there was no significant difference in the expression of miR-29b (Fig. 1B). When we compared two miRNAs *(miR-29a* and miR-29c) after normalization to the expression of *RNU48, miR-29a* was more abundantly expressed in both normal and cancer tissues.

### Effects of restoring miR-29a on cell proliferation, migration, and invasion in cervical SCC cell lines

**Figure 2.**
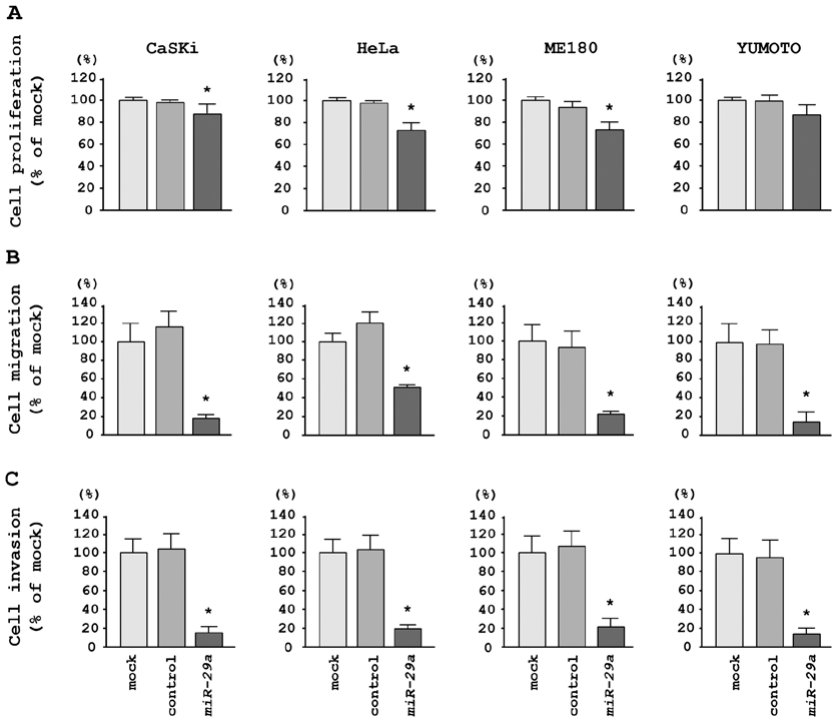
Effects of *miR-29a* restoration on the proliferation, migration and invasion of cervical cancer cell lines (CaSKi, ME180, HeLa and Yumoto). (A) Proliferation activities of cervical cancer cell lines as determined by XTT assays. *P<0.0167. (B) Migration activities of cervical cancer cell lines as determined by migration assays. *P<0.0167. (C) Invasion activities of cervical-cancer cell lines as determined by Matrigel invasion assays. *P<0.0167.

To investigate the functional effects of *miR-29a,* we performed gain-of-function studies using miRNA transfection in four cervical cancer cell lines. XTT assays demonstrated that cell proliferation was significantly inhibited in *miR-29a* transfectants in comparison with mock- or miR-control-transfected CaSKi, HeLa and ME180 cells; no inhibition was observed in Yumoto cells in this assay (Fig. 2A). We observed the following changes in proliferation, expressed as a percentage of mock-transfected cells: i) CaSKi, mock, 100.0±8.2%; miR-control, 97.9±6.6%; *miR-29a,* 87.6±5.9%; P=0.0032; ii) HeLa, mock, 100.0±7.5%; miR-control, 97.6±4.1%; *miR-29a,* 73.8±4.8%; P<0.0001; iii) ME180, mock, 100.0±4.5%; miR-control, 93.4±5.9%; *miR-29a,* 75.1±4.7%; P<0.0001, and iv) Yumoto, mock, 100.0±8.0%; miR-control, 99.9±10.6%; *miR-29a,* 85.0±9.9%; P= 0.0287 (Fig. 2A).

Migration assays demonstrated that *miR-29a* transfection significantly inhibited cell migration compared with mock- or miR-control-transfected cells. We observed the following changes in migration activity, expressed as a percentage of mock-transfected cells: i) CaSKi, mock, 100.0±14.0%; miR-control, 116.1+19.3%; *miR-29a,* 31.7±5.7%; P<0.0001; ii) HeLa, mock, 100.0±11.3%; miR-control, 124.0±14.8%; *miR-29a,* 55.3±10.6%; P<0.0001; iii) ME180, mock, 100.0±12.4%; miR-control, 89.8±16.8%; *miR-29a,* 20.3±6.4%; P<0.0001; iv) Yumoto, mock, 100.0±8.1%; miR-control, 95.4±15.8%; *miR-29a,* 13.0±6.3%; P<0.0001 (Fig. 2B).

Matrigel invasion assays demonstrated that cell invasion was significantly inhibited in *miR-29a* transfectants in comparison with mock- or miR-control-transfected cells for all cell lines tested. We observed the following changes in invasion activity, expressed as a percentage of mock-transfected cells: i) CaSKi, mock, 100.0±12.9%; miR-control, 103.4±6.3%; *miR-29a,* 16.6±7.5%; P<0.0001; ii) HeLa, mock, 100.0±16.5%; miR-control, 102.7±14.6%; *miR-29a,* 31.8+13.2%; P<0.0001; iii) ME180, mock, 100.0±22.7%; miR-control, 109.5±37.6%; *miR-29a,* 23.8±6.6%; P<0.0001; iv) Yumoto, mock, 100.0±11.9%; miR-control, 90.9±10.1%; *miR-29a,* 5.9±3.3%; P<0.0001 (Fig. 2C).

### Identification of miR-29a-regulated putative target genes

**Figure 3.**
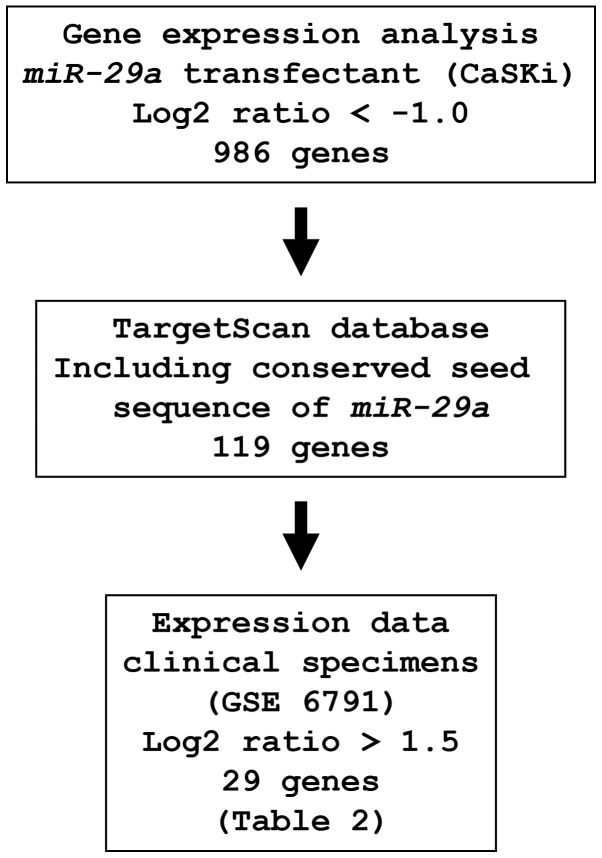
Identification of *miR-29a* regulated molecular targets. A total of 986 genes were identified by the *miR-29a* transfection into CaSKi cells. Among them, 119 genes have *miR-29a* target sites in their 3′UTR. A total of 29 genes were upregulated in cervical SCC clinical specimens by using expression data of GEO database (accession nos. GSE6791).

To identify genes regulated by *miR-29a,* we used *in silico* and genome-wide gene expression analyses. The strategy for the selection of *miR-29a*-target genes is shown in Fig. 3. First, to gain further insight into which genes were affected by *miR-29a,* we performed genome-wide gene expression analysis using *miR-29a*-transfected CaSKi cells; 986 genes were identified as downregulated (log_2_ ratio <-1.0) by *miR-29a* transfection. Next, we used the TargetScan database to examine whether these genes contained *miR-29a* binding sequences in their 3′UTRs. Finally, the gene set was analyzed with a publicly available gene expression data set in the GEO (accession no. GSE6791), and genes upregulated (log_2_ ratio >1.5) in cervical SCC were chosen. A total of 29 genes were candidate *miR-29a*-regulated oncogenic genes in cervical SCC (Table II).

As a result of our selection strategy, we identified *HSP47* as one of the most highly upregulated genes in cervical SCC tissues and one of the most significantly downregulated genes in *miR-29a*-transfected cells.

### HSP47 was directly regulated by miR-29a

**Figure 4.**
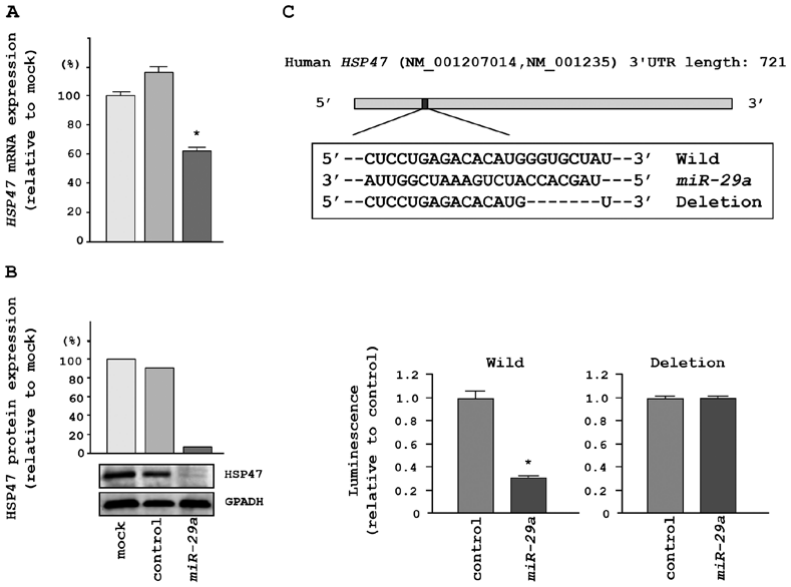
*miR-29a* directly regulates *HSP47* in HeLa cells. (A) mRNA expression of *HSP47* as measured by qRT-PCR. *GUSB* was used as an internal control. *P<0.0167. (B) Expression of HSP47 protein as measured by western blot analysis. GAPDH was used as a loading control. (C) A putative *miR-29a* binding site in the 3′UTR of *HSP47* mRNA was identified using the TargetScan database. Luciferase reporter assays were performed using a vector encoding the partial sequences of the 3′UTR containing the putative *miR-29a* target site. The vector (5 ng) and 10 nM *miR-29a* or *miR-control* were cotransfected into HeLa cells. Renilla luciferase activity was measured 24 h after transfection. The results are normalized to firefly luciferase values. *P<0.05.

We performed qRT-PCR and western blotting in HeLa cells to investigate whether HSP47 expression was reduced by restoration of *miR-29a.* The mRNA and protein expression levels of *HSP47* were significantly repressed in *miR-29a* transfectants in comparison with mock- or miR-control-transfected cells (Fig. 4A and B).

To determine whether the 3′UTR of *HSP47* mRNA had an actual target site for *miR-29a,* we performed luciferase reporter assays using a vector encoding the 3′UTR of *HSP47* mRNA. We found that the luminescence intensity was significantly reduced by transfection with *miR-29a* and the vector carrying the wild-type 3′UTR of *HSP47,* whereas transfection with a deletion vector blocked the decrease in luminescence (Fig. 4C).

### Effects of silencing HSP47 on cell proliferation, migration, and invasion in cervical SCC cell lines

**Figure 5.**
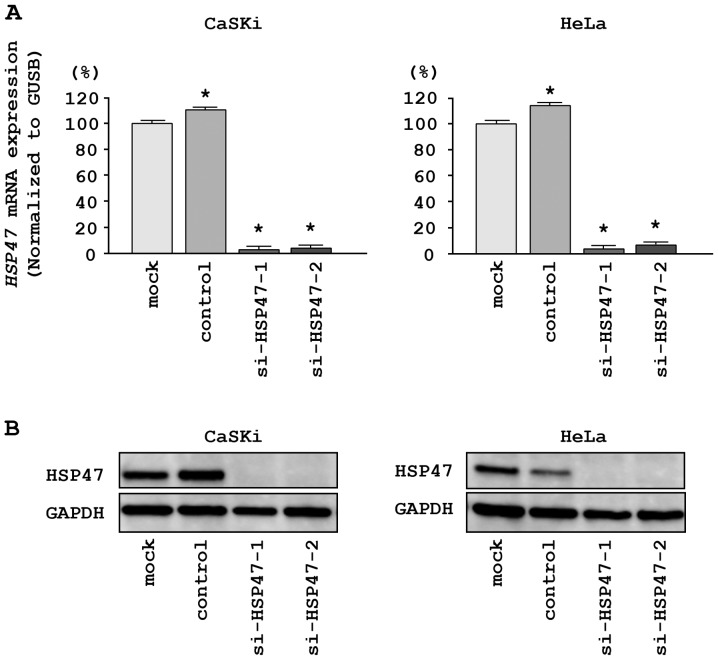
HSP47 mRNA and protein expression levels were suppressed by *si-HSP47* transfection in CaSKi and HeLa cells. (A) Expression of *HSP47* mRNA as revealed by real-time qRT-PCR. *P<0.0083. (B) Expression of HSP47 protein as revealed by western blot analysis. GAPDH was used as a loading control.

To investigate the functional role of HSP47, we performed loss-of-function studies using si-T/SWZ-transfected CaSKi and HeLa cells. First, we evaluated the knockdown efficiency of *si-HSP47* transfection. The expression of *HSP47* mRNA was repressed in two *si-HSP47* transfectants as compared with mock and si-control transfectants (P<0.0001; Fig. 5A). Moreover, the expression of HSP47 protein was repressed in *si-HSP47*1-1 and *si-HSP47-2* transfectants as compared with mock and si-control transfectants (Fig. 5B). These results showed that the two siRNAs were effective for loss-of-function assays in this study.

**Figure 6.**
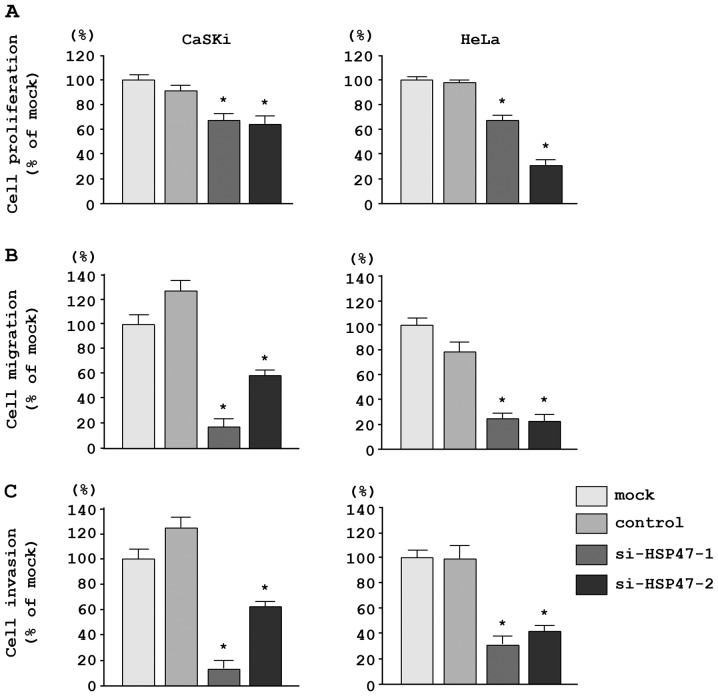
Effects of *HSP47* knockdown by *si-HSP47* transfection in CaSKi and HeLa cells. (A) Cell proliferation activities in CaSKi and HeLa cells as measured by XTT assays. *P<0.0083. (B) Cell migration activities in CaSKi and HeLa cells. *P<0.0083. (C) Cell invasion activities in CaSKi and HeLa cells. *P<0.0083.

In CaSKi and HeLa cells, XTT assays revealed significant inhibition of cell proliferation following transfection with the two different siRNAs targeting *HSP47* as compared with the growth of mock- and si-control-transfected cells. The following changes in growth were observed, expressed as a percentage of control proliferation: i) CaSKi, mock, 100.0±4.7%; miR-control, 87.0±5.7%; *si-HSP47-*1, 66.4±5.5%; *si-HSP47-*2, 63.1±7.8%; ii) HeLa, mock, 100.0±8.5%; miR-control, 100.7±8.7%; *si-HSP47-*1, 65.9±7.4%; *si-HSP47-*2, 33.0±8.7% (Fig. 6A).

Moreover, migration assays demonstrated that cell migration was significantly inhibited in CaSKi and HeLa cells transfected with the two different *si-HSP47* constructs. The following changes in migration were observed, expressed as a percentage of control migration: i) CaSKi, mock, 100.0±13.7%; miR-control, 129.8±9.9%; *si-HSP47-*1, 17.1±7.4%; *si-HSP47-*2, 57.7±5.3%; ii) HeLa, mock, 100.0±8.1%; miR-control, 74.3±9.8%; *si-HSP47-*1, 24.5±3.5%; *si-HSP47-*2, 22.9±4.7% (Fig. 6B).

Matrigel invasion assays demonstrated that cell invasion was significantly inhibited in CaSKi and HeLa cells transfected with the two different *si-HSP47* constructs. The following changes in invasion were observed, expressed as a percentage of control invasion: i) CaSKi, mock, 100.0±24.5%; miR-control, 128.0±18.9%; *si-HSP47-*1, 13.3+13.8%; *si-HSP47-*2, 63.3±24.9%; ii) HeLa, mock, 100.0±13.1%; miR-control, 97.8±17.4%; *si-HSP47-*1, 32.3±9.7%; *si-HSP47-*2, 43.2±5.2% (Fig. 6C).

### Immunohistochemistry of HSP47 in a tissue microarray

**Figure 7.**
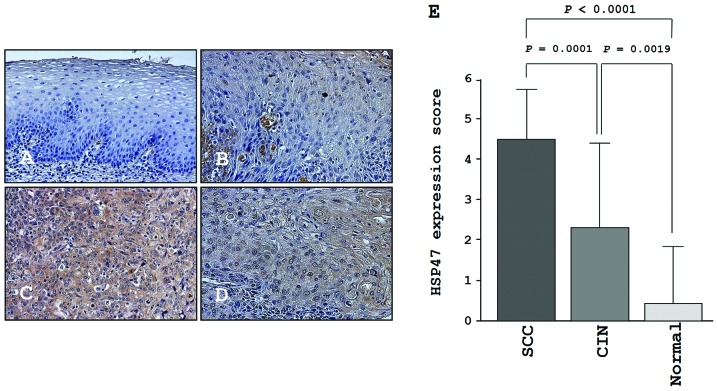
Immunohistochemical staining of HSP47 in SCC (n=30), CIN (n=10), and normal cervical tissue (n=20) by tissue microarray. (A) Negative staining in normal cervical tissue; (B) a weakly stained tumor lesion; (C) a strongly stained tumor lesion; and (D) a moderately stained tumor lesion. (E) Quantification of HSP47 expression. The expression of HSP47 was upregulated in cervical SCC specimens compared with CIN and normal cervical tissue (P<0.0001).

We confirmed the expression levels of HSP47 in normal cervical tissues, CIN tissues, and cancer tissues by immunohistochemical staining. Very low expression of HSP47 was observed in normal tissues (Fig. 7A). In CIN, weak expression of *HSP47* was observed (Fig. 7B). In contrast, HSP47 was more strongly expressed in several tumor lesions compared to normal tissues and CIN tissues (Fig. 7C and D). The expression score of HSP47 in cervical SCC was significantly higher than that in CIN (P=0.0001) and normal tissues (P<0.0001; Fig. 7E).

## Discussion

Aberrant expression of the *miR-29* family miRNAs has been reported in several types of human cancers; however, the expression status varies according to the cancer type. Decreased expression of the *miR-29* family has been observed in cholangiocarcinoma, nasopharyngeal cancer, non-small cell lung cancer, hepatocellular carcinoma, malignant peripheral nerve sheath tumors and mantle cell lymphoma. In contrast, upregulation of the *miR-29* family was reported in breast cancer, colon cancer and acute myeloid leukemia (16). In cervical cancer, it was reported that *miR-29* targets the HPV-related gene (17). In this study, our data demonstrated that *miR-29a* was significantly downregulated in cervical SCC clinical specimens and cell lines, regardless of the type of HPV infection. Furthermore, restoration of *miR-29a* in cervical cancer cells inhibited cancer cell migration and invasion, suggesting that *miR-29 a* functions as a tumor suppressor and may contribute to metastasis in cervical SCC.

The molecular mechanism through which *miR-29a* is silenced in cervical SCC is still unknown. Analysis of the promoter region of *miR-29* family miRNAs in the human genome has revealed that the *miR-29b-1/miR-29a* promoter region contains two putative E-box sites (MYC-binding sites), a Gli-binding site and four NF-KB-binding sites (18). Moreover, increased expression of the *MYC* oncogene silences *miR-29b-1/miR-29a* expression and NF-κB signaling, which is known to be activated in inflammation-related cancers, and directly represses *miR-29b-1/miR-29a* promoter activity (19). Thus, it will be necessary to identify the transcription factors that contribute to the silencing of the *miR-29* family in cervical SCC. Although the *miR-29b-1/miR-29a* and *miR-29b-2/miR-29c* formed cluster miRNAs are located within the same chromosomal regions, and share transcriptional units, the expression of *miR-29b* was not reduced in cancer tissues compared to normal tissues in this study. The reason for this is not yet clear, and further elucidation of the molecular mechanisms controlling the expression of *miR-29* family miRNAs in cancer cells is necessary.

MiRNAs are unique in their ability to regulate many protein coding genes. Bioinformatic predictions have indicated that miRNAs regulate >30% of protein coding genes (20). Aberrant expression of miRNAs causes destruction of tightly regulated miRNA/protein-coding RNA networks in human cancer cells. Therefore, identification of aberrantly expressed miRNA-mediated cancer pathways and target genes is the first step in elucidating the role of miRNAs in human cancers.

According to gene expression data and *in silico* database analysis, a total of 29 genes were selected as candidate *miR-29 a* targets. Previous reports have indicated that the *miR-29* family plays a dominant role in the regulation of extracellular matrix (ECM) genes. Indeed, luciferase reporter assays demonstrated that *miR-29a* directly targeted *HSP47,* a collagen-binding, heat-inducible glycoprotein. This is the first report demonstrating that *HSP47* was regulated by tumor-suppressive *miR-29a* in cervical SCC.

*HSP47,* a 47-kDa heat-shock protein, was first identified in fibroblasts (21) and is located within the human chromosome 11ql3.5 region, which is frequently amplified in several types of human cancers, including cervical SCC (22). Moreover, HSP47 is localized in the endoplasmic reticulum, a cellular organelle involved in the intercellular processing and secreting of procollagens (23). Many studies have demonstrated that HSP47 is overexpressed in fibrotic diseases, including kidney fibrosis, pulmonary fibrosis, cardiac fibrosis, and liver cirrhosis (24). Fibrosis is a common disease of organ dysfunction and is closely associated with ECM proteins, such as collagens, actins and fibronectins (25). Interestingly, members of the *miR-29* family have been shown to be involved in regulating ECM proteins and multiple studies have indicated that aberrant expression of *miR-29* family members contributes substantially to the development of disease (26). Thus, down-regulation of the *miR-29* family and dysregulation of HSP47 and ECM components are key events contributing to the pathogenesis of diseases, suggesting that these molecules are potential therapeutic targets.

Overexpression of HSP47 has been reported in pancreatic cancer, gastric cancer, and head and neck squamous cell carcinoma (27–29). Our present data also support previous reports, suggesting that silencing of *miR-29a* caused overexpression of *HSP47* and was an important event in the pathogenesis of cervical SCC, contributing to cancer cell migration and invasion in particular. The epithelial-to-mesenchymal transition (EMT) is a fundamental biological process whereby epithelial cells lose their polarity and undergo a transition to a mesenchymal phenotype (30). Initiation of the EMT requires external signals, such as epidermal growth factor (EGF), fibroblast growth factor (FGF), hepatocyte growth factor (HGF), and transforming growth factor-β (TGF-β) (31). The TGF-β pathway is a prominent inducer of the EMT and expression of the *miR-29* family has been shown to have an inverse relationship with the TGF-β pathway. Restoration of *miR-29* family members directly suppresses TGF-β1 and TGF-β2 and disrupts the expression of ECM proteins (32). Furthermore, ECM molecules, including collagen type I, promote the EMT through integrin and discoidin domain receptor-1 signaling (33,34). Thus, the understanding of molecular pathways and targets regulated by the tumor-suppressive *miR-29* family may provide new insights into the EMT process in cervical SCC and facilitate the development of more effective strategies for future therapeutic interventions for this disease.

In conclusion, downregulation of *miR-29 a* is a frequent event in cervical SCC. Moreover, tumor-suppressive *miR-29a* directly regulates *HSP47,* a molecular chaperone involved in the maturation of collagen molecules. Restoration of *miR-29a* or silencing of *HSP47* inhibited cancer cell migration and invasion, suggesting that the *miR-29a-HSP47* pathway contributes to the metastasis of cervical SCC. Identification of molecular targets regulated by tumor-suppressive miRNAs will provide insights into the potential mechanisms of cervical SCC oncogenesis and metastasis, facilitating the development of novel therapeutic strategies for the treatment of this disease.

## Figures and Tables

**Table I.** Characteristics of cervical SCC specimens and non-cancer specimens.

Cervical SCC specimens					

Patient no.	Age	FIGO stage	Tumor size (cm^2^)	Lymph node metastasis	HPV status
1	58	IIB	1.7×1.9	−	16
2	64	IIB	No data	−	16
3	37	IIB	3.5×3.0	+	16
4	41	IB2	8.3×3.3	−	16
5	39	IB1	3.5×3.4	−	16
6	34	IB1	3.2×2.2	−	16
7	43	IB2	4.0×8.0	−	18
8	56	IIIB	3.0×3.1	+	16, 18
9	77	IIB	3.0×2.7	−	16
10	62	IB1	3.0×2.0	−	16
11	56	IIIA	4.5×2.2	+	16
12	56	IIA	4.0×4.0	−	16
13	60	IB1	4.0×4.0	−	16
14	32	IIB	6.0×3.0	+	16
15	38	IB2	6.8×4.6	+	16
16	44	IB1	3.5×2.2	−	16
17	40	IB1	3.0×2.0	−	16
18	63	IB1	2.7×2.4	−	16

Non-cancer specimens		

Patient no.	Age	HPV status
1	44	-
2	77	-
3	75	-
4	45	-
5	47	-
6	69	-
7	40	-
8	48	-
9	41	-
10	41	-
11	34	-

**Table II.** Candidate target genes regulated by *miR-29a*.

Expression (log_2_ ratio)				
CSCC clinical specimen	*miR-29a* transfectant	Entrez gene ID	Symbol	Gene name
7.32	−3.37	871	HSP47	Heat shock protein 47
4.75	−3.04	4678	NASP	Nuclear autoantigenic sperm protein
4.69	−1.67	10951	CBX1	Chromobox homolog 1
3.29	−1.68	144455	E2F7	E2F transcription factor 7
3.08	−2.25	4291	MLF1	Myeloid leukemia factor 1
3.07	−4.27	55920	RCC2	Regulator of chromosome condensation 2
2.91	−1.50	23186	RCOR1	REST corepressor 1
2.77	−4.11	3300	DNAJB2	DnaJ (Hsp40) homolog, subfamily B, member 2
2.64	−2.66	3655	ITGA6	Integrin, α6
2.63	−2.97	79017	GGCT	γ-glutamylcyclotransferase
2.47	−1.31	8936	WASF1	WAS protein family, member 1
2.26	−1.26	4140	MARK3	MAP/microtubule affinity-regulating kinase 3
2.10	−1.15	54851	ANKRD49	Ankyrin repeat domain 49
1.91	−1.56	9949	AMMECR1	Alport syndrome, mental retardation, midface hypoplasia and elliptocytosis chromosomal region gene 1
1.87	−1.85	22877	MLXIP	MLX interacting protein
1.85	−1.49	8894	EIF2S2	Eukaryotic translation initiation factor 2, subunit 2β, 38 kDa
1.85	−1.40	3927	LASP1	LIM and SH3 protein 1
1.84	−1.42	54107	POLE3	Polymerase (DNA directed), ε3 accessory subunit
1.80	−1.24	4361	MRE11A	MRE11 meiotic recombination 11 homolog A (S. *cerevisiae*)
1.79	−1.56	7328	UBE2H	Ubiquitin-conjugating enzyme E2H
1.75	−4.69	3915	LAMC1	Laminin, γl (formerly LAMB2)
1.67	−1.57	80829	ZFP91	Zinc finger protein
1.65	−2.31	8527	DGKD	Diacylglycerol kinase, δ 130 kDa
1.61	−3.07	4232	MEST	Mesoderm specific transcript
1.60	−2.75	7168	TPM1	Tropomyosin 1 (α)
1.59	−1.62	9618	TRAF4	TNF receptor-associated factor 4
1.56	−1.28	23380	SRGAP2	SLIT-ROBO Rho GTPase activating protein 2
